# *Motherhood and Me (Mom-Me)*: The Development of an Acceptance-Based Group for Women with Postpartum Mood and Anxiety Symptoms

**DOI:** 10.3390/jcm11092345

**Published:** 2022-04-22

**Authors:** Victoria A. Grunberg, Pamela A. Geller, Kelley Durham, Alexa Bonacquisti, Jennifer L. Barkin

**Affiliations:** 1 Center for Health Outcomes and Interdisciplinary Research, Massachusetts General Hospital/Harvard Medical School, Boston, MA 02114, USA; vgrunberg@mgh.harvard.edu; 2 Department of Psychology, Drexel University, Philadelphia, PA 19104, USA; kelldurham@gmail.com; 3 Department of Obstetrics & Gynecology, Drexel University College of Medicine, Philadelphia, PA 19129, USA; 4 Graduate Counseling Psychology Department, Holy Family University, Philadelphia, PA 19114, USA; abonacquisti@holyfamily.edu; 5 Department of Community Medicine, Mercer University School of Medicine, Macon, GA 31207, USA; barkin_jl@mercer.edu

**Keywords:** postpartum mood and anxiety disorders, postpartum depression, maternal functioning, parenting, group intervention

## Abstract

Untreated postpartum mood and anxiety disorders (PMADs) place women and their families at risk for negative biopsychosocial sequelae. Innovative and tailored treatments are needed to address potential disruptions in maternal functioning. Third-wave cognitive–behavioral approaches, including acceptance and commitment therapy (ACT) and dialectical behavioral therapy (DBT), hold promise for optimizing functioning given the focus on values-based living, rather than symptom reduction. Purpose: The purpose of this paper is to describe the development of an innovative psychotherapy group for women with symptoms of PMADs. Methods: This seven-session group, *Motherhood and Me (Mom-Me)*, includes selected skills training from ACT, DBT, and Emotion-Centered Problem-Solving Therapy. Results: *Mom-Me* group sessions are described, and an outline of key information (session goals, content, and homework assignments) is provided to facilitate practical implementation. Conclusion: In line with third-wave approaches, this group was developed to enhance maternal functioning, which, in turn, may help women cope with psychological distress during the transition to motherhood.

## 1. Introduction

The postpartum period is associated with hormonal, psychosocial, and life changes, which increase women’s risk for developing postpartum mood and anxiety disorders (PMADS) [1]. About 15% to 20% of women of childbearing age worldwide experience postpartum depression [2], with high co-morbidity between depression and anxiety (40–66%) [3]. Risk factors for PMADs include a history of depression, medical complications during childbirth, limited emotional and social support, marital discord, poverty, substance abuse, and maternal chronic illness [4]. Treatments for PMADS can vary depending on symptom severity. They can include psychiatric medications, evidence-based psychotherapy, peer support groups, and self-care and healthy lifestyles [5]. Untreated PMADs have been associated with adverse personal and public health consequences for mothers, children, and families. Women with PMADS have more difficulty interacting with their infants which can, in turn, contribute to more developmental impairments among children (e.g., increased irritability, sleeping issues, and poorer language) [6,7,8]. These children are at risk for long-term cognitive, behavioral, and emotional deficits that can last through adulthood [9]. In addition, partners of women with PMADs are at increased risk of developing depression (24–50%) [10].

PMADs symptoms (e.g., depressed mood, worry, intrusive thoughts) conflict with women’s expectations of motherhood as a joyous experience that comes naturally [1]. This tension can contribute to feelings of guilt, shame, and grief [11]. These feelings affect women’s perceptions of their ability to be a “good mother” and live consistently with their parenting values [12]. This complex emotional experience coupled with family, societal, and cultural pressures can influence women’s functioning, leading to problems such as prioritizing child and family needs above their own self-care, values, and identity [13]. As such, mothers have reported feeling too emotionally and physically exhausted to take care of their own health [14]. Given the multiple roles, responsibilities, and pressures that women must juggle, it is important to empower them and help them overcome unnecessary feelings of guilt that are impeding their own self-care. Effective evidence-based treatment approaches tailored to the unique experience of postpartum women are needed to promote maternal functioning, as well as reduce PMADs symptoms.

### 1.1. Gaps in Current Evidence-Based Treatments

In addition to psychiatric medication, there are several empirically supported psychological treatments for PMADs (subclinical and clinical). Cognitive Behavior Therapy (CBT), Behavioral Activation, and Interpersonal Psychotherapy (IPT) are the current evidence-based approaches for treating PMADs. Treatments are typically short-term (6–20 sessions), delivered in individual or group formats, and conducted in a variety of settings [8,15]. Although these interventions are considered “gold-standard” treatments for postpartum women, they are limited in several ways [8]. First, few good-quality trials (only two trials out of 20) exist and the interventions have primarily been tested in the context of small pilot studies [16]. In addition, these trials tend to include White, middle-class women with a history of depression and exclude women with co-morbid diagnoses—which is not generalizable to this population [16]. There has also been large variability in the delivery method, structure, and primary targets of these interventions. For example, Cooper and colleagues’ CBT intervention [17] involved a form of interaction guidance with infants, whereas Appleby and colleagues [18] addressed lack of support and pleasurable activities. When treatment targets vary, this can limit effectiveness when interventions are applied to a variety of settings [19]. In fact, NIH has recently emphasized the importance of focusing on key mechanisms of change across interventions [19]. Additionally, most trials utilized self-report assessments of depression, rather than interviews or other outcomes measures relevant to maternal outcomes [12,16]. Finally, about 10–40% of women in these clinical trials do not exhibit improvements in depression [15], and overall, effect sizes are small [16]. Together, findings suggest that the “gold-standard” interventions for postpartum women are limited in efficacy and effectiveness and overlook important aspects of this population—which researchers have argued is why it is important to develop interventions in the context of real-world settings [16]. Innovative, tailored interventions that address the unique needs of this population and can be generalized more readily are warranted.

### 1.2. Need for Innovation

With the US Preventive Services Task Force’s 2016 report recommending universal postpartum depression screening and the Patient Protection and Affordable Care Act (2010) mandating that insurance companies cover screening [20], more women with PMADs are being identified and referred for care [21]. As recently as 2020, the United States Surgeon General submitted a Call to Action—a rare step, reserved for the most serious public health crises—to improve evidence-based care for women before, during, and after pregnancy [22]. This report indicated that Black and American Indian/Alaska Native women are disproportionally impacted by maternal mortality and morbidities (2–3 times more likely than women of other racial and ethnic groups) [22]. These initiatives are important because they highlight several current issues. First, with increased screening for postpartum depression, more women will be identified and require treatment. Next, current treatments for are not addressing these issues and new approaches are needed. Finally, women who identify as Black and/or American Indian/Alaska Native are at greatest risk for PMADS and therefore treatments need to target more diverse and generalizable populations. Given the urgency of perinatal health, new strategies are needed to enhance outcomes for these women and families.

With increased screening and the high prevalence of PMADS, the accessibility and generalizability of psychosocial care needs to be considered. To increase access, group-based approaches are cost-effective and efficient ways to provide care to many women at one time. Women have even expressed a preference for group interventions [23]. Group programs offer positive benefits, such as emotional, informational, and social support. These forms of support are important for normalizing the challenges and self-doubt new mothers often experience and can help decrease isolation [24]. Focusing on optimal functioning and flexible delivery can help overcome barriers to participation. Women of color experience more barriers to participation—may help to explain why clinical trials primarily include White, middle-class samples—including those related to logistical and sociocultural factors [25]. Stigma and cultural beliefs about motherhood, limited access to health insurance, and a lack of childcare, transportation, and employment responsibilities have all been shown to prevent women of color from participating in or completing CBT or IPT interventions (typically 12–16 sessions needed for any effect) [25]. Therefore, interventions that focus on promoting maternal functioning may help reduce the stigma associated with postpartum treatment. Increased flexibility in the format (e.g., virtual, offer support resources) may be key for improving the reach of these interventions. To enhance generalizability, interventions need to be developed for women who are experiencing health and/or psychiatric co-morbidities. The term “PMADS” was coined given the overlap of depression and anxiety symptoms, and it is more prevalent among women experiencing stressful life events and/or medical complications during pregnancy [22]. In 2018, ~7% of women who gave birth in the U.S. had gestational diabetes, ~13% of women ages 18–39 had chronic hypertension, and pregnancy complications (e.g., traumatic and/or preterm births) have only increased during COVID-19 [22,26]. Given the complexity of the perinatal period, interventions need to be more accessible and reach more diverse women.

New approaches for PMADS interventions are needed to: (1) address the stigma associated with distress after birth (“should be happy”), (2) increase accessibility, (3) overcome barriers to participation and retention, and (4) target the multiple co-morbidities that occur during the postpartum period. To help overcome each of these issues, it may be valuable for interventions to: (1) focus on resiliency and promote maternal functioning (rather than reducing distress), (2) use group formats in real-world settings, (3) be flexible in the format and delivery, and (4) use skills that provide evidence for efficacy in diverse populations (e.g., medical populations). These techniques may help reach treatment non-responders, improve efficacy and effectiveness, and promote women’s participation in psychological care.

### 1.3. Integrating Approaches for Perinatal Population

Given gaps in current gold-standard interventions, a tailored approach to intervention development is necessary to promote clinical utility. Recently, third-wave behavioral approaches, including acceptance and commitment therapy (ACT) and dialectical behavioral therapy (DBT), have shown promise for postpartum women [12,27,28]. ACT focuses on promoting functioning, rather than reducing symptoms, and is effective in diverse populations (e.g., women of color and medical populations) [27]. It is centered around flexibility and can be applied across settings and contexts [29]. DBT is valuable for managing emotions and interpersonal relationships [30]. New mothers experience changing interpersonal dynamics; therefore, communication skills are critical. In addition to these third-wave approaches, Emotion-Centered Problem-Solving Therapy (EC-PST) [31] may be helpful for this transition. EC-PST has been efficacious for medically complex populations and teaches problem-solving skills to manage daily hassles [31]. Given that women often experience co-morbidities and daily stressors, problem-solving skills are essential. Each of these approaches include skills that are of great relevance to postpartum women. Below, the relevance of ACT, DBT, and EC-PST for PMADS are described.

#### 1.3.1. Acceptance and Commitment Therapy

ACT is based on functional contextualism and relational frame theory, meaning it focuses on how people interact with their context [29]. It differs from CBT in that it focuses on the function and context of thoughts and feelings rather than the content [29]. The goal of ACT is to increase psychological flexibility, through six processes: acceptance, cognitive defusion, self-as-context, present-moment awareness (i.e., mindfulness), values, and committed action [29]. As opposed to symptom reduction, the goal is to create a meaningful life by promoting valued behavior change and optimal functioning. ACT has been effective for treating depression, anxiety, psychosis, chronic pain, and behavioral health conditions [32,33]. The ACT model is considered transdiagnostic, meaning it can treat a wide range of diagnostic presentations—which is relevant for women with PMADS given their co-morbidities [34]. More recently, ACT for PMADs has gained momentum. ACT among perinatal women [12,27,28] has demonstrated both symptom reduction and improvements in quality of life. Women with PMADS also tend to experience intrusive thoughts (e.g., thoughts about the baby being harmed), which can lead to rituals (e.g., checking behaviors) [35]. Increased attempts to control these thoughts or engage in behaviors to get rid of them can further exacerbate them [36]. ACT helps manage these difficult thoughts, as it helps women create distance from these thoughts through cognitive defusion techniques [37]. In addition, ACT is effective for parents—psychological flexibility has been linked to higher levels of responsiveness and parental adjustment [38]. ACT has also been found to be effective at reducing self-stigma in contexts where social discrimination is common, including among women of color, those with substance use difficulties, and people struggling with their sexual identity [39]. Taken together, ACT is well-suited for women as they adjust to motherhood. It may help them to enhance functioning within their maternal role, connect with values, and explore multiple identities.

#### 1.3.2. Dialectical Behavioral Therapy

DBT is based on dialectical philosophy—the understanding that multiple truths exist [40]. DBT combines strategies of behavior therapy with Eastern mindfulness practices, including a worldview that emphasizes the synthesis of opposites [40]. Within this approach, therapists strive to balance acceptance with change-oriented strategies [40]. DBT was initially developed for complex and difficult-to-treat mental health disorders, such as borderline personality disorder, which is more prevalent among women [30]. DBT has been adapted to treat binge eating, depression, and suicide ideation [40]. DBT includes skills such as mindfulness, acceptance, distress tolerance, emotion regulation, and interpersonal effectiveness. DBT directly addresses domains that contribute to depression within the postpartum period, including difficulty coping with distress (distress tolerance) and emotions (emotion regulation), focusing on the present (mindfulness), and managing interpersonal relationships and social supports (interpersonal effectiveness) [41]. Mindfulness has been shown to help prevent depression among postpartum women and promote skillful parenting [42]. DBT is helpful across the lifespan (adolescence to adulthood), which is important for this population given that postpartum distress occurs across all ages, especially younger women [42]. DBT skills help address communication challenges, emotional lability, and interpersonal difficulties, which are inherent barriers to optimal functioning during the postpartum period. Given that PMADs has implications for the entire family, these skills are particularly relevant. 

#### 1.3.3. Emotion-Centered Problem-Solving Therapy

Emotion-Centered Problem-Solving Therapy (EC-PST) promotes effective coping with daily hassles and significant stressors, thereby improving physical and mental health outcomes [31]. EC-PST has been effective among those with depression, anxiety, diabetes, pain, and post-traumatic stress disorder [31]. This approach is applicable because it has been effective in treating trauma and medical impairments—two issues that are common in women with perinatal distress. Nezu and colleagues [31] reported that treatment goals include adopting an adaptive worldview towards problems (e.g., optimism and positive self-efficacy) and implementing problem-solving behaviors. Mothers face barriers to meeting goals (e.g., time restraints, childcare demands, balancing work with family) and EC-PST offers skills that can address these everyday problems to improve maternal functioning. Together, EC-PST, coupled with ACT and DBT, could enhance maternal functioning by improving family communication and interactions and providing effective problem-solving skills to cope with everyday stressors.

## 2. A New Intervention: *Motherhood and Me (Mom-Me)*

To address limitations in current psychological treatments for postpartum women, skills from ACT, DBT, and EC-PST were integrated to develop the *Motherhood and Me (Mom-Me)* group. *Mom-Me* is focused on promoting maternal functioning (rather than reducing depression and anxiety). The goal was to make the intervention applicable to real-world settings. *Mom-Me* was developed for diverse women with subthreshold and clinically significant symptoms of PMADs who were enrolled in a perinatal intensive outpatient treatment program in Philadelphia, PA, *Mother Baby Connections* (MBC) [21]. Because this group was developed in the context of MBC, the targeted population was accessible and available to participate. MBC includes women of diverse racial, ethnic, and economic backgrounds because it helps overcome important barriers to care. MBC services are free of cost, childcare is available by trained professionals onsite, transportation options or payments are provided (as needed), and therapists are flexible if women need to step out briefly to manage employment or child-related issues. At MBC, women attended two full days of intensive outpatient treatment, including individual therapies (psychological, medication management), dyadic therapies (mother–baby interaction therapy), and group therapies (e.g., psychoeducational, skills-based, yoga, and creative art therapy) [21]. Details on this program have been published previously [21]. *Mom-Me* was one component of MBC that brought mothers together to learn adaptive skills to improve maternal functioning. The goals of *Mom-Me* include: (1) teach skills to improve maternal functioning; and (2) facilitate social support among mothers to normalize experiences. Given that it was developed in the context of this program (real-world clinical setting), it has not yet been formally tested as a stand-alone treatment. The purpose of this paper is to describe the development of the *Mom-Me* group in the context of an intensive outpatient treatment program. Below, the rationale and content for each session of *Mom-Me* are described.

### 2.1. Mom-Me Group Format

*Mom-Me* was developed as a weekly 50 min group for women with postpartum depression and anxiety symptoms. This format was chosen because new mothers prefer group-based intervention and benefit from the normalization and validation of their experience [43]. The group empowers mothers to help each other by offering peer support and practicing effective communication skills in sessions. The facilitator also encourages discussion and social support among mothers. This group was developed in the context of a larger clinical service (MBC). The MBC program is embedded within the Psychological Services Center at Drexel University and offered Wednesdays and Fridays from 9–3 pm. The *Mom-Me* group was delivered by one facilitator (MBC had 2–3 doctoral-level psychological graduate students who rotated delivering it) from 12–1 pm on Wednesdays. *Mom-Me* typically included 3–6 women/group because MBC serves six mothers at a time and women tend to be discharged at different times (after 8–16 weeks of treatment). *Mom-Me* used an open-group format, allowing members and number of people to change from week to week. This flexibility helps account for the changing composition of group members and enhance inclusivity. Given the number of barriers that these women experience (e.g., transportation, employment, and childcare), it was important to make the group accessible [25]. Seven sessions were chosen given that this allowed for the rotation of topics. Women who had been in the program longer and already participated in a prior session were able to share their experiences with a particular skill with moms newer to the group. While the sessions were designed in an intentional order, each can stand-alone. Prior research and clinical expertise helped to inform the session order [12,21]—described within each session below. However, the stand-alone format allowed for flexibility regarding session order and number of sessions offered. Together, the open-group, stand-alone format was intentionally chosen to increase accessibility and promote participation. Mothers already report excessive guilt [11], so this format was used to prevent these feelings and emphasize provider acceptance of the real-world challenges that new mothers experience.

### 2.2. Mom-Me Group Content

Regarding the content, it developed by clinical psychologists within MBC (including the Co-Founder/Co-Director) who have research and clinical expertise in perinatal mental health. The content was informed by prior research [12,21] and clinical expertise working with this population. Sessions integrated core skills from ACT, DBT, and EC-PST, as indicated by the intervention developers [29,31,40]. Sessions addressed the following skills: values (ACT), present-moment awareness (ACT), understanding emotions (ACT/DBT), emotion regulation (DBT), acceptance (ACT/DBT), interpersonal effectiveness (DBT), and planful problem-solving (EC-PST). Sessions included psycho-education, experiential exercises, and solicitation of examples from mothers’ lives. Table 1 presents information on how to conduct the sessions, including the topics, treatment modality, discussion content and group activities, and sample home practice handouts. For each session, the rationale, goals, tools, scripts, and examples from de-identified *Mom-Me* group are described below.

#### 2.2.1. Values (Session 1)

Clarifying values helps individuals identify what is important to them. In ACT, the goal is to engage in behaviors that are values-consistent to live a meaningful life [29]. A mother is often consumed by her new child and may struggle to balance motherhood with other values [13]. In fact, women have reported losing their individual identity as they transition to motherhood, but also report lacking confidence in this new role [46]. Given the daily hassles and difficult balance of motherhood, career, and relationships, values can provide perspective by directing focus on what is truly important (e.g., time spent with baby). The concept of values was chosen as the first session because they help provide both direction and motivation for therapy [44]. Therefore, skills taught later could be linked to values to promote motivation and buy-in.

The goals for this session are to provide psycho-education on values and help *Mom-Me* participants identify 1–3 personal values. First, the facilitator describes the distinction between values and goals (e.g., values are what you want life to stand for and help direct your life whereas goals are milestones that can be achieved). Then, participants are provided with tools (i.e., Values Clarification and Values Compass worksheets) [44] to help them identify top values. The facilitator encourages discussion about the value of “being a good mother”, self-care, and adult interaction by asking questions such as: “What does being a good mother mean?” and “Are you spending your time in a way that is in line with your definition of self-care?”. The facilitator leads a discussion on barriers to values-consistent behavior, how to overcome these barriers, and the role of stigma when balancing values. Participants frequently report barriers such as lack of childcare support, guilt, and concern about child’s safety without them. Before ending, mothers identify specific goals for the next week that will help them live in line with their values (e.g., “call a friend on Tuesday after baby’s nap”).

#### 2.2.2. Present-Moment Awareness (Mindfulness; Session 2)

Present-moment awareness (mindfulness) means paying attention, on purpose and without judgment, to the present moment [44]. Present-moment awareness serves different functions including: (1) developing awareness of internal experience, and (2) increasing attention and purpose in activities. Mindfulness-based techniques have been effective in reducing depression and anxiety among postpartum women [47]. Present-moment awareness promotes mothers’ full engagement in meaningful activities, such as spending time with their babies and families [48]. This skill increases awareness of internal experiences of distress, and ultimately, acceptance. Present-moment awareness helps mothers to identify self-critical thoughts and notice when self-judgments are based on societal or internal expectations of their mothering ability [12]. Given that this skill helps patients to identify emotions and thoughts [47], it was taught prior to emotion-oriented sessions.

The goals for this session are to promote increased awareness and curiosity towards internal experiences. The facilitator leads a discussion about stressors specific to motherhood. Participants have shared that they find themselves stressed about new things such as feeding and sleeping schedules, not having time to shower, not seeing friends, and managing co-parenting responsibilities. Then, the facilitator provides psycho-education on this skill by explaining how it can improve moms’ ability to manage stress, focus on positive moments with their baby, and avoid “autopilot mode” by paying attention (with intention) to the present moment. The facilitator leads a mindful eating or visualization exercise (~5 min). Participants are provided with a Mindfulness of Breath worksheet [45] and identify barriers that may interfere with practice over the next week. Mothers help each other problem-solve barriers such as logistics, time, stigma, and skepticism.

#### 2.2.3. Understanding Emotions (Session 3)

This session is focused on the value of emotions and the functions they serve. Within DBT, the function of each emotion to promote a greater understanding of emotions. For example, sadness often indicates loss, the need to grieve, and is associated with an urge to isolate [40]. The pressure of motherhood as a time of joy and happiness invalidates the experience of negative emotions—sadness, grief, and shame. It was important to teach this skill prior to the emotion regulation session. The goal was to help women understand the purpose of emotions and validate emotional experiences before regulating them.

The goals of this session are to discuss the function of emotions and to normalize the range of emotions experienced during motherhood. The facilitator provides psycho-education on emotions (i.e., complex and have an important purpose). The facilitator helps participants identify the function of each emotion and their associated thoughts and action urge (using white board). Participants have endorsed a desire to “hide emotions” around their babies because they fear their negative emotions will interfere with infant development or upset their child. The facilitator illustrates the detriment of pushing away emotions through a metaphor (i.e., Beach Ball metaphor) [29]. The facilitator encourages mothers to share their experiences trying to “push away” or “not notice” emotions (because they want to put on a happy face and be strong). Mothers appreciate discussions on why it is valuable for children to observe a range of emotions throughout development. It is important to allow mothers to experience emotions, challenge their perceptions of “good” and “bad” emotions, and normalize the range of emotions. Participants are encouraged to identify emotions over the next week using the Emotions handout [40].

#### 2.2.4. Emotion Regulation (Session 4)

This session can help mothers learn strategies to manage high levels of arousal or strong emotions. It is based on the DBT Emotion Regulation toolkit [40], which uses cognitive and behavioral techniques to manage strong emotions. It is important for women to have tools to manage emotions like anger, sadness, and shame. This session was purposely taught after the emotions session so that women were more in tune with their emotions, especially in the context of their families [42].

The goal of this session is to help manage high levels of arousal or strong emotions. The facilitator explains that when elevated emotions can interfere with goals and values. Then, participants are provided with the DBT Emotion Regulation toolkit [40] as a tool to highlight different ways to manage arousal. Regulation techniques include observing and accepting emotions (e.g., mindfulness and acceptance), checking the facts (e.g., identifying if the emotion fits the facts of the situation), and opposite action (e.g., being active when sad, instead of isolating) [40]. The facilitator encourages discussion on when these skills can be used, and participants provide examples from their own lives. For example, several participants have endorsed the value of opposite action when with in-laws given the frustration they often experience. The facilitator highlights that regulation does not mean suppression or avoidance of emotions. Participants are encouraged to practice an emotion regulation skill over the next week.

#### 2.2.5. Acceptance (Session 5)

This session draws on the core ACT principle of experiential acceptance and the DBT distress tolerance skill of reality acceptance. This session promotes the acceptance of both internal experiences (i.e., thoughts and emotions) and external circumstances that cannot be changed (e.g., loss of expected birth experience). A core principle in ACT is learning to accept thoughts and emotions because they are part of the human experience and cannot be controlled. According to Hayes and colleagues [29], the goal of experiential acceptance is to embrace internal experiences, instead of attempting to control them, to live a richer and more meaningful life. Reality acceptance means accepting the facts of one’s life, the limitations of one’s future, and that life is worth living even when it is painful [30]. Reality acceptance is important during the transition to motherhood because of the significant changes to relationships and everyday life. This skill was taught after present-moment awareness and emotion-related sessions because awareness of thoughts and emotions is an important first step to acceptance [29].

The goals of this session are to promote the acceptance of internal experiences and external circumstances. The facilitator asks mothers what the term acceptance means to them. Psycho-education on acceptance is provided: “acceptance is not giving up or throwing in the towel, it is an ongoing process of living your life fully without trying to change or control your emotions, thoughts, or external events” [12]. The facilitator introduces the Tug-of-War with the Monster metaphor from ACT [29], using both internal and external experiences to demonstrate the “monster”. *Mom-Me* participants “monsters” often include thoughts about being a “bad mom”, difficult partners, or health issues. The facilitator explains that “control is the problem” given that the human experience is filled with experiences that cannot be controlled. Participants are encouraged to consider times when they attempted to control emotions—typically involve isolating from family or inhibiting emotions in front of their babies. The facilitator explains that experiential acceptance means “embracing internal experiences” and “create space” from them to live in line with values. Participants are encouraged to engage in *reality acceptance*—accept unchangeable aspects of their lives. Mothers are encouraged to review the Accepting Emotions handout [37] for homework.

#### 2.2.6. Interpersonal Effectiveness (Session 6)

Interpersonal effectiveness skills from DBT help individuals attend to relationships, manage priorities and demands, balance “wants” with “shoulds”, and build a sense of mastery and self-respect [30]. Mothers tend to minimize their own needs and desires, especially shortly after childbirth [49]. It is useful for mothers to communicate their needs to partners, set boundaries with in-laws or family members, and learn to negotiate. These skills are valuable as children grow and mothers need to impart clear expectations when parenting. Given the need for awareness and emotion regulation for improved communication, it was taught after those sessions [50].

The goal of this session are to teach mothers effective ways to communicate in relationships. The facilitator introduces the topic: “As you all know, being a mother means managing relationships and communicating with others. It seems most of the time is spent figuring out how to manage relationships with babies, partners, in-laws, and friends”. Participants share their experiences communicating needs to loved ones. The facilitator uses the Interpersonal Effectiveness Skills handout [30] as a tool to share communication strategies (i.e., DEAR MAN, GIVE, and FAST). Over the next week, mothers are encouraged to apply this tool to a difficult interpersonal situation, such as requesting childcare assistance or advocating for self-care with a family member.

#### 2.2.7. Planful Problem-Solving (Session 7)

Planful problem-solving is the core skill of EC-PST [31]. Within this framework, real-life problem solving, or social problem solving, is defined as the self-directed process by which individuals attempt to identify and/or develop adaptive coping solutions for problems, both acute and chronic, encountered in daily life [31]. This tool involves identifying the problem, developing concrete goals, determining barriers, generating creative solutions, assessing solutions, and creating and implementing an action plan [31]. New mothers face a variety of everyday problems. Planful problem-solving can help problems seem more manageable and enhance rational and optimistic problem-solving [31]. This session was taught last given the value of learning coping skills prior to problem-solving [31]. Because mothers tend to focus on their family [13], it is important to have them spend time on themselves before teaching communication and problem-solving strategies.

The goals of session 7 are to introduce the planful problem-solving skill and apply it to a real-world example. The facilitator introduces idea of “problem-solving” and notes that although it can seem intuitive, we often do it too quickly and without much thought. The facilitator explains that: “Planful problem-solving is a tool that helps us to access the rational part of brain (as opposed to emotion center) so we can determine the most effective ways to overcome barriers and meet goals. We will walk through each step together using a shared problem related to motherhood”. Using the Planful Problem-Solving worksheet [31] and a white board, the facilitator helps participants identify a shared problem related to motherhood and then outline a specific, barriers, creative solutions, and action plan. Examples of problems and goals identified during groups include: finding enough time with partners (goal: increase positive communication this week), balancing baby with self-care (goal: take 30 min each day to do something for self), and balancing motherhood with other responsibilities (goal: ask for help from partner at least twice over the next week). *Mom-Me* participants enjoy outlining the steps together as it facilitates creative thought, collaboration, and the normalization of everyday problems. Participants have reported that it is “empowering” to creatively solve a complex social problem. The facilitator encourages participants to apply the action plan or use the steps to address a new problem over the week.

## 3. Clinical Implications and Future Directions

*Mom-Me* is an innovative acceptance-based group intervention that incorporates ACT, DBT, and EC-PST. It was developed to enhance maternal functioning among diverse women with varying severity levels of postpartum distress in a real-world clinical setting (MBC). Given that the group was conducted at MBC, the targeted population was accessible and interested in participating. Future researchers and clinicians may consider advertising groups through social media, hospitals, mother–baby programs, and community providers to ensure that the group is accessible. Values, present-moment awareness, emotions, emotion regulation, acceptance, interpersonal effectiveness, and planful problem-solving were selected as session topics based on empirical research and clinical expertise [12,27,28,51]. Although the group does not aim to reduce depressive and anxiety symptoms, it aims to improve maternal functioning—which is likely to decrease psychological distress and improve quality of life. The purpose of *Mom-Me* is to introduce skills that women can apply in their daily lives to cope with postpartum distress. This paper provides rationale for the format and content of this group as well as practical goals and tools that clinicians can consider for practice. Clinicians are encouraged to use the information as they see fit to inform their own practice and/or groups for postpartum women. *Mom-Me* can be flexibly integrated into inpatient or outpatient clinical settings. Table 1 describes key components of each session; however, clinicians can tailor the content and handouts to their patients’ needs—focusing on patient-specific values, problems, or communication strategies. Regarding format, *Mom-Me* is best delivered by 1–2 facilitators in the context of a group (3+ mothers) given the importance of normalization and social support. It was developed as a seven session, 50 min clinical protocol, however, each session can be stand-alone and abbreviated (~30 min) if needed. Clinicians are encouraged to adapt the content and format for their practice. Future research will test the feasibility and acceptability of *Mom-Me* to optimize format and content. To enhance retention, it is important for clinicians to consider flexible delivery options. For example, they may offer groups in settings where childcare services are available, allow babies to be present as needed, or offer virtual groups. Open-format groups—mothers can “drop in”—may also help improve engagement as it is less burdensome and may decrease feelings of guilt if they miss a session. It is also important to offer cost-effectiveness options (e.g., sliding scale) to increase access. Clinicians may consider having trainees conduct groups as it would promote their training and avoid costly services for mothers. Future work will explore how flexible this group can be while also maintaining fidelity to treatment content. 

Because the *Mom-Me* group is currently being delivered within MBC [21], this intervention was unable to be tested as a stand-alone group. Future work will iterate, refine, and test *Mom-Me* for feasibility, and ultimately, efficacy–effectiveness, consistent with the NIH Stage Model of intervention development [52,53]. The first step will include qualitative interviews and/or focus groups with perinatal women and providers to determine the unique needs of this population (using the DART framework) [54]. Given the barriers to participation due to COVID-19 and parenting-related barriers [25], participants will be asked about their preference for in-person, virtual, or hybrid delivery options. Depending on interview findings, *Mom-Me* may be adapted for an entirely virtual platform (over live video). Next, an open pilot will be conducted (*N* ≤ 5 per NIH recommendations) [52] and mothers will be asked to complete measures and exit interviews. Assessment measures will include the Barkin Index of Maternal Functioning (BIMF) [55,56,57] and Edinburgh Postnatal Depression Sale (EPDS) [58] to examine changes in maternal functioning (primary outcome) and PMADS symptoms (secondary outcome) at baseline, post-test, and follow-up (3–6 months later). The BIMF—which has strong reliability (Cronbach’s alpha ≥ 0.8) and been well-validated among postpartum women—includes seven functional domains (i.e., self-care, infant care, mother–child interaction, psychological well-being, social support, responsibility manage, and adjustment) [55,56,57]. The EPDS—which has strong reliability (Cronbach’s alpha ≥ 0.8) and validity (sensitivity = 0.8; specificity = 0.9)—is the most widely used measure of postpartum distress [58]. These assessments will be gathered by MBC volunteer staff (undergraduate or master students) who are not involved in group delivery. This open pilot will explore initial feasibility and acceptability (participation, retention, completion of measures, credibility, satisfaction). Findings will help iterate and refine *Mom-Me*, which will then be tested for feasibility and acceptability. Once *Mom-Me* is iterated based on Stages 0 and 1 of the NIH Stage Model [52], MBC Directors will be formally tested for feasibility and acceptability (*N* ≥ 50 per NIH recommendations) [52] and ultimately efficacy–effectiveness trials in future trials. The goal is to develop an accessible, group-based intervention that can be adapted to various settings, including telehealth delivery. Beyond the in-person treatment restrictions imposed by the pandemic, delivery through an online format may help overcome barriers to participation. Given the theoretical and clinical value of *Mom-Me,* future work also is needed to determine how *Mom-Me* can be adapted for private practice, outpatient clinics, and obstetrics/gynecology settings with co-located psychosocial providers.

## 4. Conclusions

About 15–20% of women experience mood and anxiety symptoms postpartum [3], with potential for sustained distress. PMADS symptoms can negatively impact functioning, which has direct and indirect implications for mothers, infants, and families [10]. This paper described an innovative acceptance-based group intervention, *Mom-Me*, that was developed in the context of an intensive outpatient treatment program—MBC [21]. The goal of this clinically informed group was to improve maternal functioning. The group combines skills from ACT, DBT, and EC-PST to help women notice internal experiences, behave consistently with values, problem-solve stressors, and communicate effectively. Given the value of peer support, evidence-based skills, and acceptance-based approaches, this group holds promise for informing perinatal-focused treatment approaches. Clinicians and researchers can use this group to inform and enhance new psychotherapy approaches for women coping with postpartum distress.

## Figures and Tables

**Table 1:** Session outline of the “*Motherhood and Me (Mom-Me)*” group.

Session	Modality	Skill	Content	Sample Handout
1	Acceptance and Commitment Therapy	Values	Provide psycho-education on values vs. goals Group activity on identifying personal values Discuss relevance to being a “good mother” and self-care. Discuss barriers to engaging in behaviors in line with values. Identify person-specific goals consistent with values.	Values Compass [44]
2	Acceptance and Commitment Therapy	Present-Moment Awareness (Mindfulness)	Discuss unique stressors and “lack of time” for moms. Provide psycho-education on mindfulness. Conduct autopilot worksheet individually and discuss relevance to motherhood as part of a group discussion. Practice present-moment awareness as a group (e.g., eating).	Mindfulness of the Breath [45]
3	Dialectical Behavioral Therapy	Understanding Emotions	Provide psycho-education on variety of emotions. Discuss the function and neutrality of all emotions. Facilitate discussion on emotions in context of motherhood.	Emotions handout [40]
4	Dialectical Behavioral Therapy	Emotion Regulation	Review prior session content—value and function of emotions. Group discussion on difficult or unhelpful emotions, especially in relationships with partner, baby, in-laws, and other family members. Apply emotional regulation skills to personal examples provided by mothers (i.e., checking facts, opposite action, and positive events).	Opposite Action worksheet [40]
5	Acceptance and Commitment Therapy & Dialectical Behavioral Therapy	Acceptance	Facilitate discussion on complexity of the human experience. Provide psycho-education on acceptance in ACT and DBT. Mothers offer examples of challenges they need to accept. Discuss coping patterns and acceptance as an alternative.	Accepting Emotions [29]
6	Dialectical Behavioral Therapy	Interpersonal Effectiveness	Discuss the importance of relationships to motherhood. Discuss ways of communicating needs to family members. Present DEARMAN approach to family communication. Group discussion on the importance of communicating needs to loved ones (i.e., self-care, asking for help, and expressing needs to partner/family).	“DEARMAN” worksheet [40]
7	Emotion- Centered Problem Solving Therapy	Planful Problem- Solving	Provide psycho-education on problem-solving (and the relevance to the amygdala and prefrontal cortex). Group discussion on problems and how they were solved. As group, apply skills to one common motherhood problem. Provide psycho-education and practice planful problem-solving steps as a group (i.e., define problem, barriers, creative solutions, pros and cons solutions, action plan)—use whiteboard. Discuss importance of being flexible in roles and relationships.	Planful Problem-Solving worksheet [31]

## Data Availability

Not applicable.
